# miR-MaGiC improves quantification accuracy for small RNA-seq

**DOI:** 10.1186/s13104-018-3418-2

**Published:** 2018-05-15

**Authors:** Pamela H. Russell, Brian Vestal, Wen Shi, Pratyaydipta D. Rudra, Robin Dowell, Richard Radcliffe, Laura Saba, Katerina Kechris

**Affiliations:** 1 Department of Biostatistics and Informatics, Colorado School of Public Health, Aurora, CO 80045 USA; 2 Computational Bioscience Program, University of Colorado, Aurora, CO 80045 USA; 3 Department of Molecular, Cellular, and Developmental Biology, University of Colorado, Boulder, CO 80309 USA; 4 Department of Pharmaceutical Sciences, University of Colorado Skaggs School of Pharmacy and Pharmaceutical Sciences, Aurora, CO 80045 USA; 5 Center for Genes, Environment and Health, National Jewish Health, Denver, CO 80206 USA

**Keywords:** MicroRNA, miRNA, Small RNA-seq, Expression quantification

## Abstract

**Objective:**

Many tools have been developed to profile microRNA (miRNA) expression from small RNA-seq data. These tools must contend with several issues: the small size of miRNAs, the small number of unique miRNAs, the fact that similar miRNAs can be transcribed from multiple loci, and the presence of miRNA isoforms known as isomiRs. Methods failing to address these issues can return misleading information. We propose a novel quantification method designed to address these concerns.

**Results:**

We present miR-MaGiC, a novel miRNA quantification method, implemented as a cross-platform tool in Java. miR-MaGiC performs stringent mapping to a core region of each miRNA and defines a meaningful set of target miRNA sequences by collapsing the miRNA space to “functional groups”. We hypothesize that these two features, mapping stringency and collapsing, provide more optimal quantification to a more meaningful unit (i.e., miRNA family). We test miR-MaGiC and several published methods on 210 small RNA-seq libraries, evaluating each method’s ability to accurately reflect global miRNA expression profiles. We define accuracy as total counts close to the total number of input reads originating from miRNAs. We find that miR-MaGiC, which incorporates both stringency and collapsing, provides the most accurate counts.

**Electronic supplementary material:**

The online version of this article (10.1186/s13104-018-3418-2) contains supplementary material, which is available to authorized users.

## Introduction

MicroRNAs (miRNAs) are endogenous small (~ 23 nt) RNA molecules that contribute to post-transcriptional regulation of target messenger RNAs (mRNAs) in plants and animals [1, 2]. In recent years, many tools have been developed to estimate miRNA expression from small RNA-seq data. These include CAP-miRSeq [3], Chimira [4], CPSS [5], iSRAP [6], miRanalyzer [7], the miRDeep2 quantifier [8], miRExpress [9], miRge [10], miRNAKey [11], mirTools [12], Oasis [13], omiRAs [14], Shortran [15], and sRNAbench [16]. Table 1 summarizes these methods. In a typical workflow, the read counts form the foundation for downstream analyses such as differential expression and co-expression analysis. Therefore, accurate expression quantification is essential for the validity of downstream results.

**Table 1:** miRNA quantification methods for small RNA-seq

Method	Year	Architecture	miRNA search space	Aligner	Alignment to miRNAs	Handling multi-mapped Reads	Counts
CAP-miRSeq [3]	2014	Pipeline for Linux environ-ment	miRBase mature and precursor	Bowtie [24]	Alignment within miRDeep2 [11]	All valid mappings reported by miRDeep2	Use miRDeep2 for counts; for mature miRNAs with multiple precursors, return weighted counts for each precursor
Chimira [4]	2015	Web application	miRBase precursors	BLASTn [25]	Max 2 mismatches	User choice to keep the first match or assign fractional counts to all matches	
CPSS [5]	2012	Web application	miRBase	SOAP2 [26]	By default, best hits with max 2 mismatches	By default, report one random alignment per read	
iSRAP [6]	2015	Pipeline for Linux environment	User defined	Bowtie2 [27]	Seed length 20; max 1 mismatch in seed	Bowtie2 default: report one best alignment per read	BEDTools [28]
miRanalyz- er [7]	2011	Web application	miRBase mature, maturestar, unobserved maturestar, and hairpin	Bowtie	Seed length 17. By default, max 1 mismatch in seed	By default, allow up to 10 mappings per read	
miRDeep2 quantifier [8]	2012	Perl script	miRBase mature and precursor	Bowtie	By default, max 1 mismatch	By default, all valid mappings	Count all instances where a read maps to the same part of precursor as a mature miRNA
miRExpress [9]	2009	Command line tools	miRBase; identical mature miRNAs collapsed	Novel aligner based on Smith–Waterman algorithm [29]	Find one best mapping per read. By default, keep those where read length is equal to miRNA length and identity is 100%	Identify at most one mapping per read	Count all valid mappings for each collapsed miRNA
miRge [10]	2015	Perl script	miRBase mature and precursor; identical mature miRNAs collapsed; near-identical families merged after alignment	Bowtie	Sequential alignments: first perfect match, then up to 2 mismatches	All valid mappings	Counts per miRNA with identical and near identical miRNAs merged together
miRNAKey [11]	2010	Java GUI with Perl backend	miRBase mature or precursor	BWA [30]	User defined max edit distance	Optionally use SEQ-EM [31] to optimize distribution of multiply aligned reads	Optimized distribution of reads to miRNAs
mirTools 2.0 [12]	2013	Web application	miRBase	SOAP2	By default, best hits with max 2 mismatches	By default, report one random alignment per read	
Oasis [13]	2015	Web application	miRBase	STAR [32]	Max mismatches 5% of read length	STAR default: report all alignments for reads with up to 10 mappings only	Feature-Counts [33]
omiRAs [14]	2013	Web application	miRBase	Bowtie	Max 2 mismatches, best stratum only	Assign fractional counts for multiply mapped reads	
Shortran [15]	2012	Command line modules	miRBase	Bowtie	User defined		
sRNAbench [16]	2014	Web application	User defined	Bowtie	User defined	User defined; by default, allow up to 10 mappings per read	Two output files: one with all mappings counted; one with only one mapping counted per read

Implementation details of several recently published methods for miRNA expression quantification from small RNA-seq

The effectiveness of quantification methods may be affected by three issues particular to miRNAs. One issue involves mapping accuracy. The small size of miRNA molecules leads to short sequencing reads after adapter removal. Short reads are less likely to be aligned uniquely to the genome [17]; this issue could be compounded by individual genetic variation at the endogenous locus producing the read [18]. The second issue involves challenges of functional interpretation. Identical or near-identical miRNAs are often transcribed from multiple genomic loci [19, 20]. So as not to introduce count bias, quantification methods must deal with reads that map ambiguously to multiple loci or miRNA sequences. In addition, there are many fewer unique miRNA molecules than large RNAs. Normalization methods such as total read count or quantile normalization are less robust with fewer features and highly skewed distributions. Therefore, the handling of multi-mapped reads can have a larger impact on normalized counts for miRNAs compared to larger RNAs. Third, isomiRs—miRNA variants that can be expressed in a cell type specific manner—present a challenge for mapping and functional interpretation. Research suggests that the three main classes of isomiRs (5′ isomiRs, 3′ isomiRs, and polymorphic isomiRs) may have differing functional consequences [21, 22]. The question of whether isomiRs should be counted and, if so, which ones should be merged with their parent miRNA for expression analysis, is nontrivial and should be addressed by quantification methods.

Methods that fail to adequately address these issues can return misleading quantification results. We examined the accuracy of several published methods as well as a novel quantification pipeline that incorporates stringent mapping and collapsing of the miRNA space into meaningful functional units.

## Main text

### Results

We designed a quantification method with the following objectives: (1) perform highly stringent mapping to a core region of miRNA sequences, minimizing the number of ambiguous mappings, and (2) perform collapsing to associate reads with functional classes of miRNAs instead of individual annotated miRNAs. Functional classes of miRNAs, subsequently referred to as “functional groups”, are defined by the user to be groups of miRNAs that are considered equivalent in the context of the study goals. For instance, if the study aims to address binding of target mRNAs, families of highly similar miRNAs that bind the same targets can be considered equivalent. This consideration allows reads to be counted at most once per functional group; counts are then returned at the group level. We implemented a pipeline, miR-MaGiC, that incorporates these features. For details of the software and workflow, see Additional file 1: Additional material and Figure S1.

We tested miR-MaGiC and several publicly available methods on 210 mouse brain small RNA-seq libraries. This dataset was chosen due to the large number of samples and high sequencing depth, making it a valuable test case for comparing methods, while the variability in proportion of miRNA reads between libraries provided an interesting testing scenario. We ran 7 quantification schemes for each library: iSRAP [6], the miRDeep2 quantifier [8], miRge [10], a modified version of miRge, and three collapsing conditions for miR-MaGiC. Our modified version of miRge removed its final round of alignments to mature miRNAs, a highly permissive alignment step that allowed up to two mismatches per read; we suspected that this step may introduce noise to the counts. See Additional file 1: Table S1 and Additional material.

To evaluate the methods, we reasoned that methods which correctly handle the issues particular to miRNA quantification should return total counts that reflect the number of reads originating from miRNAs in the input library. We estimated the number of miRNA reads in each library as the number of adapter-clipped reads between 19 and 23 nucleotides in length; 95% of miRNA loci and 91% of unique mature miRNAs in miRBase fall in this length range. The libraries each had between 50% and 72% of reads in this range. We examined how well each method reflected this estimated number of input miRNA reads in terms of total output read count, calculating the mean squared error between the estimated number of input miRNA reads and the output total counts. A lower score would indicate more accurate counts and therefore less distortion and bias introduced during normalization by the method-dependent total count.

**Fig. 1:**
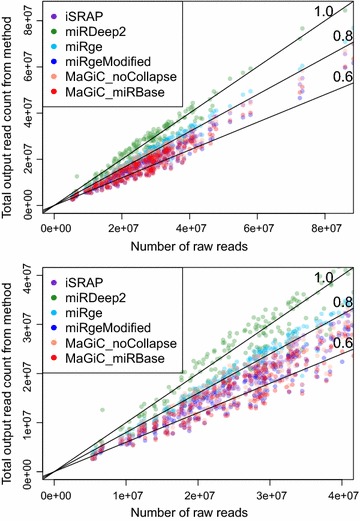
Total counts returned by each method (Top: all samples; bottom: zoom in on the area of highest density). Each dot represents one library being quantified by one method. Results for miR-MaGiC with collapsing by MIMAT number are not pictured as they are extremely similar to collapsing by miRBase name. A dot’s position along the horizontal axis indicates the number of raw reads for the library. Its position along the vertical axis indicates the total count returned for the library by the method indicated by dot color. The solid lines indicate a theoretical ratio of total count to input raw reads. For example, a dot lying on the 0.8 line would mean the total counts for that library and quantification method was 0.8 times the number of raw reads. For dots lying above the 1.0 line, the total counts for that library and method added up to more than the number of raw reads. See Additional file 1: Table S1 for detailed explanation of method abbreviations

Due to different implementation choices, the methods systematically return different levels of total absolute counts (Fig. 1). The miRDeep2 quantifier returns the highest counts because it first matches mature miRNAs to precursors in a many-to-many mapping, then counts every instance of a read matching one of these mature miRNA/precursor pairs. As expected, miR-MaGiC returns reduced total counts when functional group collapsing is performed, as opposed to no collapsing. Because the read counts for miRNAs are right skewed (Additional file 1: Figure S2), double counting in any of the highly expressed miRNAs can dramatically change the total read count. See Additional file 1: Additional material and Figure S3 for a case study of miRNAs that are treated differently by different methods.

**Fig. 2:**
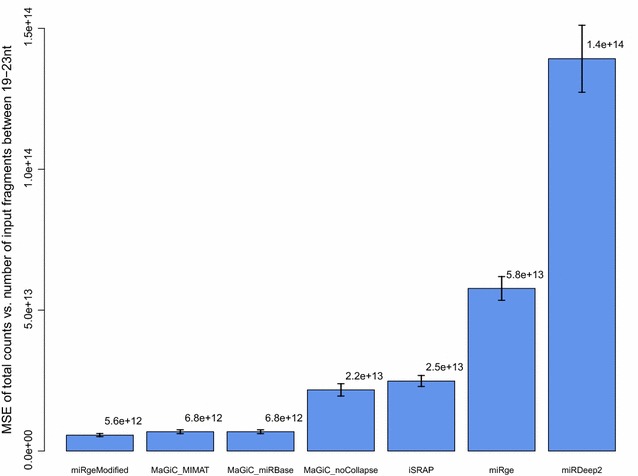
Method accuracy: total counts compared to number of input miRNA reads. Method accuracy was evaluated as the mean squared error (MSE) between the estimated number of miRNA reads used as input to the method (fragments 19–23 nt in length) and the total count derived by each quantification method. The error bars indicate ± one standard error of the mean. See Additional file 1: Table S1 for detailed explanation of method abbreviations

Comparing miR-MaGiC to published software, miR-MaGiC with collapsing by functional group showed the best accuracy (Fig. 2). The least accurate method is the miRDeep2 quantifier, probably due to double counting reads that map to multiple precursors. The closest method to miR-MaGiC is miRge, which also incorporates collapsing but uses permissive mapping. As expected, miR-MaGiC with no functional group collapsing is less accurate than with collapsing. When we modified the miRge code to remove the final round of highly permissive alignments, performance improved dramatically and the method gained a slight advantage over miR-MaGiC with collapsing. One possible explanation for why the published version of miRge is less accurate than the more stringent modified version is that the permissive alignment step allows some non-miRNA reads to be mapped to miRNAs.

### Conclusions

We have proposed a quantification method, miR-MaGiC, that addresses several issues particular to miRNAs, including their small size, low complexity, family structure, and isoforms. miR-MaGiC uses stringent mapping to reduce noise associated with the small size and low complexity of miRNAs, while allowing for uncertainty at the endpoints of reads and miRNAs. Final counts are returned at the group level instead of the individual miRNA level. Recommended group tables are provided for common species on the miR-MaGiC web page, https://github.com/KechrisLab/miR-MaGiC.

We tested miR-MaGiC as well as three published methods on a set of 210 small RNA-seq libraries. We evaluated the faithfulness of the final total counts to the original number of miRNA reads per library. Importantly, we found that methods which specifically address the above issues produced the greatest accuracy in overall counts. The novelty of miR-MaGiC is the combination of stringent mapping to a core region of each miRNA and collapsing by functional group.

To evaluate this combination of features we tested miR-MaGiC with and without collapsing, observing that collapsing in fact improves accuracy. Regarding mapping stringency, the published version of miRge, which performs collapsing, performed poorly according to our accuracy metric, but we suspected this may be due to over-permissiveness of one of its alignment steps. Once we modified this detail, miRge emerged as comparable to miR-MaGiC, with a slight advantage in accuracy. In summary, when methods use one feature but not the other (i.e., miR-MaGiC_noCollapse and miRge in Fig. 2), or neither feature (i.e., iSRAP and miRDeep2 in Fig. 2) there is a notable drop in accuracy.

Our analysis of miRge indicated that more noise than signal is introduced if methods try to capture isomiRs simply by allowing more mismatches. miR-MaGiC uses stringent mapping to reduce noise associated with the small size and low complexity of miRNAs. This decision effectively causes 5′ and 3′ isomiRs to be merged with their parent miRNA while discarding polymorphic isomiRs. 3′ isomiRs are the most common class of isomiR and are thought to be largely functionally redundant, while 5′ and polymorphic isomiRs are less common but can affect target binding [21, 22]. Therefore, miR-MaGiC merges most functionally redundant miRNA isoforms with their parent miRNA while also possibly including 5′ isoforms that may affect function. This decision has the effect of including the largest class of isomiRs which are currently believed to be largely functionally redundant while excluding polymorphic isomiRs which may have distinct functions.

### Discussion

In this work, we examined accurate quantification of miRNA expression based on sequencing. Several issues particular to miRNAs can affect the accuracy of quantification methods based on small RNA-seq. These issues include the small size of miRNAs, the low complexity of the overall repertoire of miRNAs, the fact that highly similar miRNAs can be processed from different genomic loci, and the presence of isomiRs. Furthermore, it is important that quantification be performed at an appropriate level of granularity to be functionally meaningful. Implementation choices at the quantification step can have a significant impact on common downstream steps such as normalization and interpretation of expression results. When counts are split over multiple features, the multiple testing burden is increased and statistical power is reduced. In addition, the relatively low complexity of the miRNA repertoire means that a handful of highly expressed miRNAs can have an impact on the library size used for normalization.

Our work demonstrates the importance of identifying the most meaningful unit of information when studying miRNA expression. We find that results are most accurate when we associate each read with one meaningful unit such as a miRNA family. To accomplish this, our proposed method, miR-MaGiC, looks for a stringent match to one or more members of the family and then ignores which member(s) it matched and reports results for the family. The mapping is stringent in one sense, but also flexible at the ends of each miRNA, as these can be affected by isomiRs or artifacts in the reads. The most meaningful level of granularity for a particular study may vary. We therefore recommend that investigators understand the implementation details of various quantification methods and choose a method that will return the most meaningful expression profile for their study.

### Materials and methods

#### Known miRNAs and creation of individualized miRNA sequences

We used the mouse miRNA database in miRBase version 21 [23]. See Additional file 1 for details.

#### Defining functional groups of miRNAs

Our pipeline, miR-MaGiC, counts mappings of reads to functional groups of miRNAs instead of individual miRNAs. We evaluated three different groupings of miRNAs. The first was no collapsing by functional group. The second combined miRNAs with the same miRBase accession number (“MIMAT” number) before an underscore. The final grouping combined miRNAs with the same core number, letter (if applicable), and 3p/5p identifier. See Additional file 1 for details.

#### Test with publicly available software packages

We chose publicly available methods to include in our comparison based on several criteria: (1) ability to be run in batch jobs on a Linux cluster, (2) success of installation and execution on our Linux environment, and (3) methods representing a variety of quantification strategies. These criteria led to choosing iSRAP [6], the miRDeep2 quantifier [8], and miRge [10]. 210 mouse whole brain small RNA-seq libraries were analyzed. Run details are in Additional file 1: Table S1 and Additional material.

## Limitations

Our analysis demonstrates that for short sequences from a low-complexity repertoire, a high level of mapping stringency is important for minimizing noise. However, a limitation of this high stringency is that errors in reads or individual variation in miRNAs could lead to incorrectly missed read mappings, i.e., an increase in false negative mappings. Another limitation is that miR-MaGiC only generates counts and does not perform analyses such as normalization and differential expression, in contrast to other small RNA-seq analysis tools that perform multiple analyses in a pipeline fashion. Nonetheless, the resulting miR-MaGiC quantification is easily plugged into other downstream analyses.

## Additional file

**Additional file 1.** Additional materials, methods, figures, and tables.

## Abbreviations

miRNA: microRNA
mRNA: messenger RNA
MSE: mean squared error
RPM: reads per million
