# Supplementary material for: miR-MaGiC improves quantification accuracy for small RNA-seq
Source: BMC Res Notes. 2018 May 15;11:296. doi: 10.1186/s13104-018-3418-2 (PMC5952827; doi:10.1186/s13104-018-3418-2)
Supplement: Supplementary file 1 — Additional file 1. Additional materials, methods, figures, and tables. [file 13104_2018_3418_MOESM1_ESM.docx]

**ADDITIONAL MATERIAL**

**The LXS panel of recombinant inbred mouse strains**

The long-sleep (LS) and short-sleep (SS) selectively bred lines of mice were derived from a heterogeneous stock originally generated from 8 inbred mouse strains. These lines were selected based on long or short duration of loss of righting reflex due to ethanol [[1,2]](https://paperpile.com/c/QCn3le/edanD+q6Bqr). The Inbred LS (ILS) and Inbred SS (ISS) were subsequently generated and used as founders for the LXS (ILSXISS) recombinant inbred panel [[2]](https://paperpile.com/c/QCn3le/q6Bqr).

**Sample preparation, library construction, and sequencing**

A total of 184 mice from 61 strains of the LXS panel were sacrificed and total RNA was extracted from whole brain tissue using the RNeasy Plus Universal Midi, Mini and miniElute kits (Qiagen, Valencia, CA). Small RNA libraries were prepared from these samples using the TruSeq Small RNA Sample Prep Kit (Illumina, San Diego, CA). Libraries were prepared in five batches; 17 samples were included in two or more batches for a total of 212 libraries; two of these failed in sequencing, leaving 210 for analysis. Size selection was performed to purify fragments between 20 and 35 bp in length. The libraries were sequenced on the Illumina HiSeq 2500 platform. Adapters were clipped and reads were quality filtered using the FASTX-Toolkit 0.0.13 (Gordon and Hannon. Fastx-toolkit. FASTQ/A short-reads preprocessing tools. Unpublished. http://hannonlab.cshl.edu/fastx_toolkit. 2010) with the options “-a GGAATTCTCGGGTGCCAAGG -n true -Q 33” for the fastx_clipper and “-q 20 -p 95 -Q 33” for the fastq_quality_filter. We obtained an average of ~23 million filtered reads per library. Two low-complexity libraries that produced under one million reads were considered to have failed and were removed from further analysis. All libraries had a median fragment length of 23nt after adapter clipping. The proportion of reads between 19 and 23nt — the canonical size range for miRNAs — ranged from 50% to 72%.

**Known miRNAs**

For all methods, we used the mouse miRNA database in miRBase version 21 [[3]](https://paperpile.com/c/QCn3le/FamDl). iSRAP used the 2,045 genomic annotations of miRNA loci in mm10 coordinates. miRge used an internal modified version of the 1,915 mature miRNA sequences, adding additional searchable miRNA sequences featuring each allele of validated polymorphisms. miRDeep2 used the 1,915 mature sequences and the 1,193 precursors. miR-MaGiC used the 2,045 genomic annotations of miRNA loci with strain-specific variation incorporated (see “Creation of individualized miRNA sequences”).

**Creation of individualized miRNA sequences**

miR-MaGiC calls for individualized miRNA sequences if available. Although we found genetic variation affecting expressed miRNAs to be negligible within our test dataset (Only 7 of the 2,045 miRNA loci contained an identified polymorphism in at least one strain as described below, and had a count greater than 10 in at least one library), we nevertheless recommend using target miRNA sequences that incorporate individual variation if possible, due to the high stringency of the mapping step. As our samples were from the LXS recombinant inbred panel, we were able to assume no heterozygosity and use public genotype data obtained from other animals from the same inbred strains [[2]](https://paperpile.com/c/QCn3le/q6Bqr). We estimated haplotypes in the LXS strains by combining high-density sequencing-based variant calls in the two parental strains [[4]](https://paperpile.com/c/QCn3le/k8SED) with lower density genotyping in the LXS strains [[5]](https://paperpile.com/c/QCn3le/CVsKu). Briefly, for each LXS strain and each pair of adjacent genotypes within 1Mb and 1cM of each other, we checked whether the strain genotypes matched one, both, or neither parental strain at both positions. If the LXS strain matched one parental strain at both positions, we estimated a haplotype for the LXS strain consisting of all high-density parental genotypes between the two positions. Genetic distances between chromosome positions were extracted from the Jackson Laboratory Mouse Map Converter (http://cgd.jax.org/mousemapconverter/) using the male genetic map in [[6]](https://paperpile.com/c/QCn3le/pipQb).

Dense estimated genotypes were converted to VCF format for compatibility with downstream tools. Variant coordinates on mm10 were converted to relative coordinates within overlapping miRNAs, counting from the 5’ end of the molecule. The GATK tool FastaAlternateReferenceMaker [[7]](https://paperpile.com/c/QCn3le/H4aLy) was used to convert reference miRNA sequences to strain-specific miRNA versions for each strain, incorporating the strain variants described in relative miRNA coordinates. Identical mature miRNAs at this stage were combined into a single searchable sequence. Code to reproduce the individualized miRNA sequences is available at https://github.com/KechrisLab/miR-MaGiC.

**Defining functional groups of miRNAs**

miR-MaGiC counts mappings of reads to functional groups of miRNAs instead of individual miRNAs. The functional groups are defined by the user in a table provided as input to the pipeline. We tested miR-MaGiC with three different groupings of miRNAs. Because we used individualized miRNA sequences and collapsed identical mature miRNAs, this step started from slightly different numbers of miRNAs across libraries. For simplicity, the following numbers refer to the reference (mm10) sequences. The first grouping simply placed each miRNA into its own group, so there was effectively no collapsing by functional group. At this stage, the 2,045 annotated loci in miRBase had been collapsed into 1,920 unique mature sequences; therefore, this grouping consisted of 1,920 groups. The second grouping combined miRNAs with the same miRBase accession number (“MIMAT” number) before an underscore. For example, *MIMAT0000123:mmu-miR-1a-3p* and *MIMAT0000123_1:mmu-miR-1a-3p*, which differ by one nucleotide at their 5’ and 3’ ends, were combined into one group. This grouping included 1,891 groups. The final grouping was by miRNA name. We combined miRNAs with the same core number, letter (if applicable), and 3p/5p identifier. For example, *MIMAT0017066:mmu-miR-92a-1-5p* and *MIMAT0004635:mmu-miR-92a-2-5p* were combined into a group, and *MIMAT0031401:mmu-miR-8099* and *MIMAT0031401_1:mmu-miR-8099* were combined into another group. This grouping included 1,860 functional groups. A script to reproduce the functional groups is available at https://github.com/KechrisLab/miR-MaGiC.

**miR-MaGiC workflow details**

We implemented a pipeline, miR-MaGiC, consisting of standalone Java programs organized into a Snakemake [[8]](https://paperpile.com/c/QCn3le/6Mt43) workflow. The pipeline runs from the command line on any system with a working installation of Snakemake and Java 8 or higher. Each run of the pipeline handles one library; the steps of the pipeline are (1) align the reads: for a specified *k*, identify all perfect matches of length *k* between individual clipped reads in fastq format and mature miRNAs (recommended *k*=20 to capture the functional core of the mature miRNA while allowing for uncertainty at the endpoints), and (2) summarize functional groups: if a read matches multiple miRNAs in a group, only count one mapping to the entire group, then return counts at the group level. The workflow is illustrated in Supplemental Figure 1. We note that, for miRNAs shorter than *k*, perfect matches of the length of the miRNA are sought. Because the pipeline performs stringent mapping, requiring a perfect local match between the read and a miRNA sequence, we recommend using target sequences that have incorporated individual genotypes if this information is available. Alternatively, if individual variation is not available, one could include modified versions of miRNAs incorporating known polymorphisms as in [[9]](https://paperpile.com/c/QCn3le/olQdN).

**Testing against publicly available methods**

We tested miR-MaGiC and three publicly available methods on a dataset consisting of 210 small RNA-seq libraries from mouse whole brain. Although adapter clipping is theoretically not required for the kmer-based approach of miR-MaGiC, we used clipped reads as input to all methods for consistency in the comparison. The methods tested were iSRAP [[10]](https://paperpile.com/c/QCn3le/Vol15), the miRDeep2 quantifier [[11]](https://paperpile.com/c/QCn3le/hFJOu), and miRge [[9]](https://paperpile.com/c/QCn3le/olQdN); see Table 1 for detail on these and other published methods. We ran 7 quantification schemes for each library: iSRAP, the miRDeep2 quantifier, miRge, a modified version of miRge, and three collapsing conditions for miR-MaGiC. miRge, like miR-MaGiC, also performs collapsing but has an alternative mapping approach. Our modified version of miRge removed its final round of alignments to mature miRNAs, a highly permissive alignment step that allowed up to two mismatches per read; we suspected that this step may introduce noise to the counts. Supplemental Table 1 details these 7 quantification schemes. For each method, we extracted only the raw miRNA counts and ignored any subsequent downstream analyses performed by the method. For miRge, the output file “miR.Counts.csv” was used for each library. For the miRDeep2 quantifier, the output file “miRNA_expressed.csv” was used for each library. For iSRAP, the output file “trimmed.sorted.miRNA.counts.txt” was used for each library. For miR-MaGiC, the output file “final_counts.{sample}.txt” was used for each library. (Names refer to files written by each tool.) Scripts to reproduce the analysis in the paper based on the counts are available at https://github.com/KechrisLab/miR-MaGiC. The tools were run on our x86_64 GNU/Linux cluster managed by the Platform LSF load balancer. After removing two libraries that failed in sequencing and two more libraries that repeatedly failed in the iSRAP pipeline, 208 libraries returned successful results for all 7 methods.

A comparison of run times is difficult to interpret because two of the tools (iSRAP, miRge) are pipelines that incorporate extra steps in addition to mapping and quantification. Nevertheless, average run times are reported in Supplemental Table 1.

**Case study of miRNAs that are treated differently by different methods**

Of all miRNAs that have multiple nearly identical sequences sharing the same miRBase name, *mmu-let-7f-5p* is the most highly expressed in our samples. miRBase contains two versions of this miRNA that are processed from different precursors; their annotated mature sequences differ only at their end positions (**U**GAGGUAGUAGAUUGUAUAGU and GAGGUAGUAGAUUGUAUAGU**U**). The methods that perform collapsing over functional groups (miR-MaGiC and miRge) report a single count for this family. The methods that do not perform collapsing (iSRAP and the miRDeep2 quantifier) divide the counts over the two members of the family (Supplemental Figure 3)[.](https://docs.google.com/document/d/1jTFQdkjnpHgGpoB0b8poh4p2u_7oHnsZ1N9YZo49I9I/edit#bookmark=id.yvo88jzf91ns) miR-MaGiC with no collapsing imitates a possible implementation choice to double count reads with multiple matches. This is similar to the behavior of the miRDeep2 quantifier in cases of multiple matches to precursors. *mmu-let-7a-5p* is another miRNA for which miRBase contains two versions processed from different precursors. Again, the annotated mature sequences differ only at their end positions (**U**GAGGUAGUAGGUUGUAUAGU and GAGGUAGUAGGUUGUAUAGU**U**). In this example, presumably aligners have more difficulty matching reads to one precursor or the other. Therefore, reads are more evenly divided between the two versions by iSRAP, which allows only one mapping per read, and more reads are counted multiple times by the miRDeep2 quantifier, which counts all mappings of reads to any precursor. Because the read counts for miRNAs are right skewed (Supplemental Figure 2), double counting in any of the highly expressed miRNAs, such as these two examples, could dramatically change the total read count.

**ADDITIONAL FIGURES AND LEGENDS**


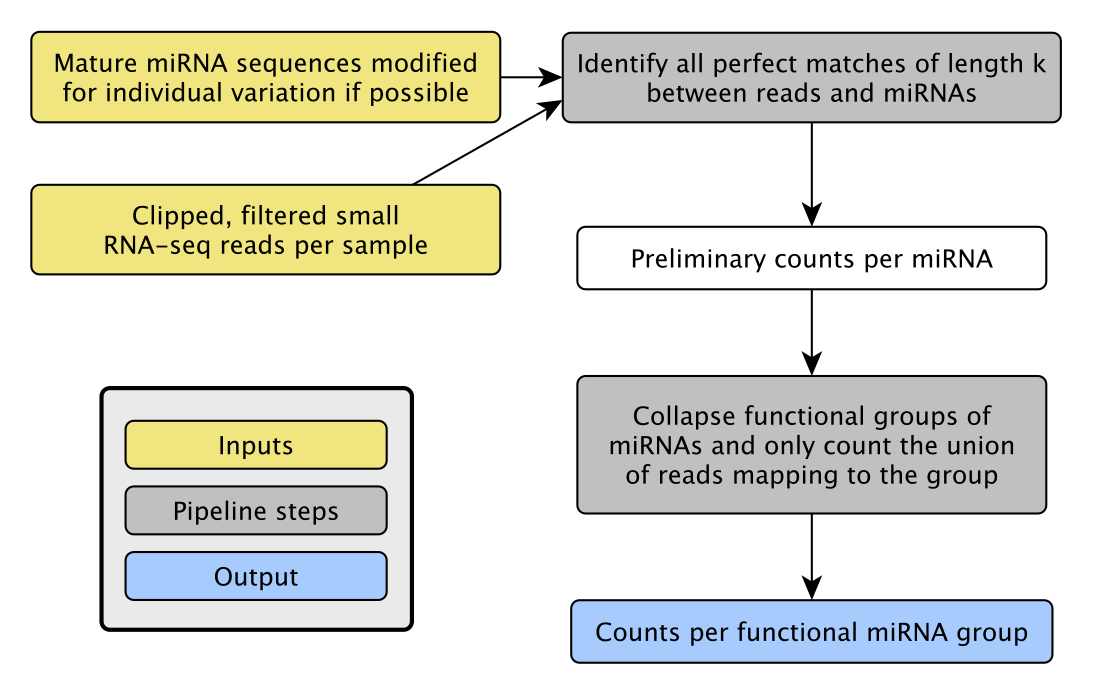


**Additional file 1: Figure S1: miR-MaGiC workflow.**


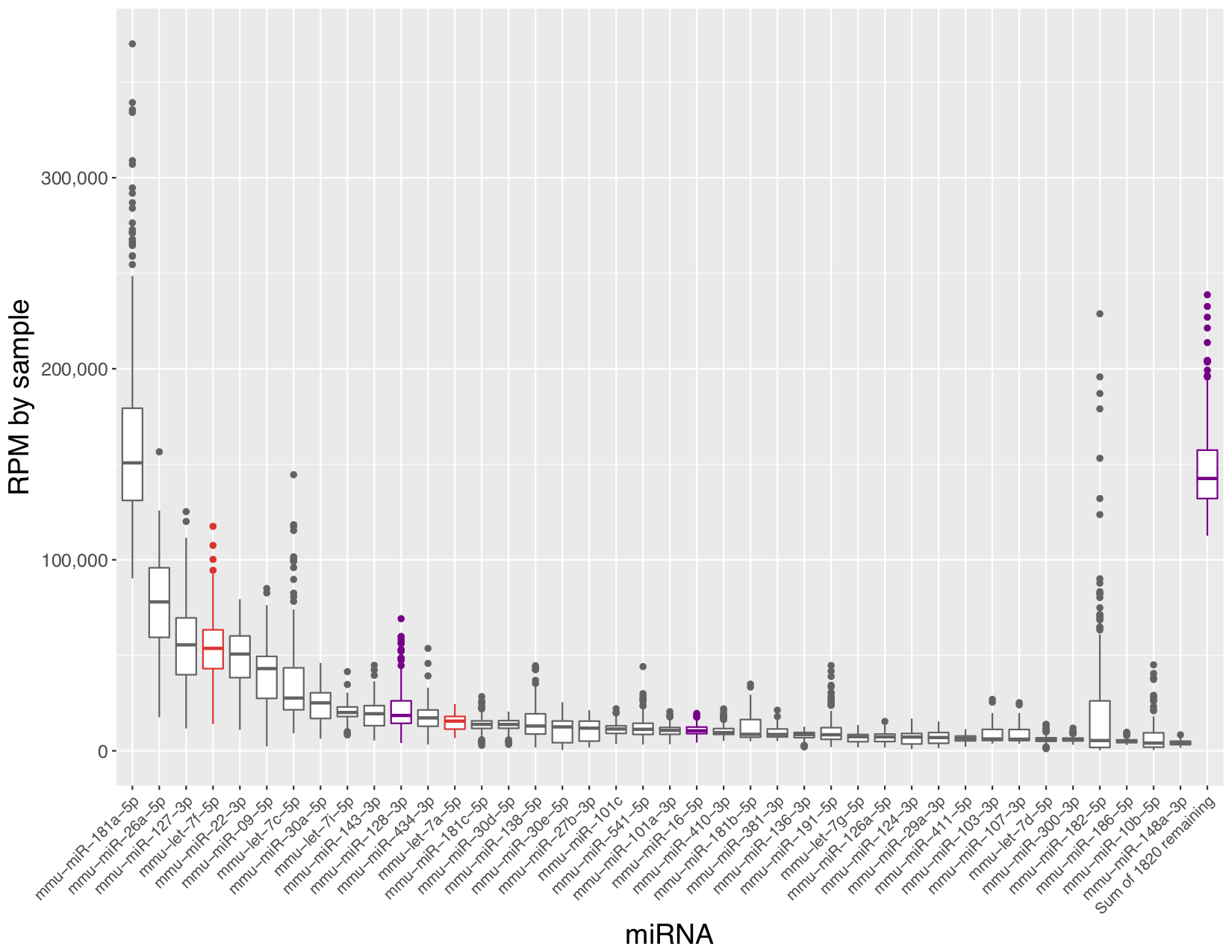


**Additional file 1: Figure S2:** **Top expressed miRNAs according to miR-MaGiC.** Reads per million (RPM) from the miR-MaGiC quantification with collapsing by miRBase name is shown. Each box corresponds to one functional group of miRNAs quantified by the method. The y-values are RPM for individual samples. The box spans the 25th through 75th percentiles. The median is represented by the horizontal line inside the box. Whisker length is at most 1.5 times the interquartile range. Points outside the span of the whiskers are plotted individually. The top 40 groups by median RPM are shown, followed by a final box for the total of the remaining groups. Red color indicates the two groups featured in Supplemental Figure 3: highly expressed miRNAs that receive different treatment by different methods. Purple color indicates additional miRNA groups consisting of multiple nearly identical miRNAs. Note that 50,000 reads per million corresponds to 5% of total counts for a library.


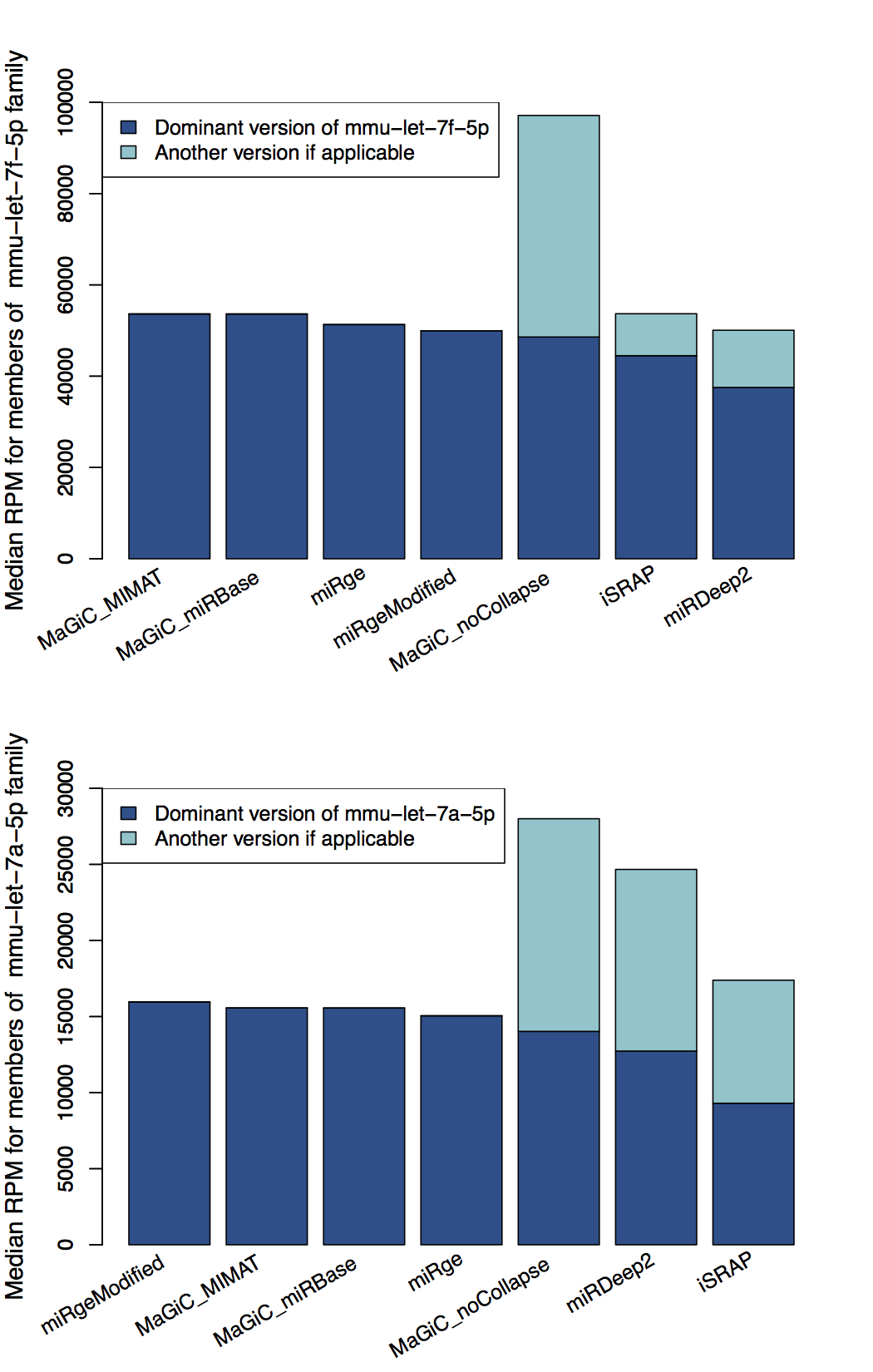


**Additional file 1: Figure S3: Collapsing to functional groups of mature miRNAs.** (Top) *mmu-let-7f-5p*, the most highly expressed functional group with multiple members in our samples. (Bottom) *mmu-let-7a-5p* is another miRNA for which miRBase contains two versions processed from different precursors. For each quantification method, the median reads per million (RPM) across libraries for this miRNA family is shown. The first four methods in the plot collapse the two similar miRNAs to one count. The last three methods divide the counts over both versions. Note that 50,000 reads per million corresponds to 5% of total counts for a library.

**SUPPLEMENTAL TABLES AND LEGENDS**

**Additional file 1: Table S1**

| **Method**  **(abbreviation for figures)** | **Program version used** | **Options used** | **Mean CPU time per sample** |
| --- | --- | --- | --- |
| iSRAP (iSRAP) [[10]](https://paperpile.com/c/QCn3le/Vol15) | 0.1 | GENOME_REF: mm10 assembly  RRNA_REF: Mus musculus rRNA sequences downloaded from RefSeq  GTF: Ensembl gene annotation downloaded on 1/20/15  BED_REFERENCE_MIRNA: miRBase version 21  INTERSECT_MINIMUM_OVERLAP_miRNA: 0.85  INTERSECT_MINIMUM_OVERLAP_smallRNA: 0.8  PICARD_TOOLS_PATH: Picard tools version 1.90  RNA_SEQC_JAR: RNA-SeQC version 1.1.7  TRIMMOMATIC_JAR: Trimmomatic version 0.36 | 61.1 minutes (note: includes Trimmomatic adapter clipping step that could not be turned off) |
| miRDeep2 quantifier (miRDeep2) [[11]](https://paperpile.com/c/QCn3le/hFJOu) | 0.0.5 | Mapper: -e -h -m -n -u  Quantifier: -d -p [miRBase 21 mus musculus hairpins] -m [miRBase 21 mus musculus mature] | 5.6 minutes |
| miRge (miRge) [[9]](https://paperpile.com/c/QCn3le/olQdN) | 2.0 | --adapter illumina --species mouse | 9.7 minutes |
| Modified miRge (miRgeModified) | Modified 2.0 | --adapter illumina --species mouse | 9.4 minutes |
| miR-MaGiC - no group collapse (MaGiC_noCollapse) | 1.0.0 | mirna: miRBase 21 mus musculus mature miRNA genomic loci with strain variation incorporated; sequences extracted from mm10; identical sequences collapsed  mirna_gp: each miRNA is in a group by itself  k: 20  plus_strand_only: True | 21.3 minutes |
| miR-MaGiC - group by miRBase accession (MaGiC_MIMAT) | 1.0.0 | mirna: miRBase 21 mus musculus mature miRNA genomic loci with strain variation incorporated; sequences extracted from mm10; identical sequences collapsed  mirna_gp: miRNAs with same MIMAT accession up to an optional underscore are in the same group  k: 20  plus_strand_only: True | 23.1 minutes |
| miR-MaGiC - group by miRNA name (MaGiC_miRBase) | 1.0.0 | mirna: miRBase 21 mus musculus mature miRNA genomic loci with strain variation incorporated; sequences extracted from mm10; identical sequences collapsed  mirna_gp: miRBase names are mapped to a group by the successive application of three regular expressions:   - s/(\-\d+[a-z]?\-)\d+\-/\1/g - s/\.2-3p//g - s/\.2-5p//g   k: 20  plus_strand_only: True | 22.3 minutes |

**Additional file 1: Table S1.** miRNA quantification methods tested. Run details for the 7 quantification schemes run on each library. miRge is the published version, which includes five sequential rounds of Bowtie alignment to various target spaces and with various alignment parameters. In modified miRge, we removed the last permissive alignment step from the source code (the mapping to mature miRNAs with flags “-f -l 15 -5 1 -3 2 -n 2”).

**Additional file 1: references**

[1. Markel PD, DeFries JC, Johnson TE. Use of repeated measures in an analysis of ethanol-induced loss of righting reflex in inbred long-sleep and short-sleep mice. Alcohol Clin Exp Res [Internet]. 1995;19:299–304. Available from:](http://paperpile.com/b/QCn3le/edanD) <https://www.ncbi.nlm.nih.gov/pubmed/7625561>

[2. Williams RW, Bennett B, Lu L, Gu J, DeFries JC, Carosone-Link PJ, et al. Genetic structure of the LXS panel of recombinant inbred mouse strains: a powerful resource for complex trait analysis. Mamm Genome [Internet]. 2004;15:637–47. Available from:](http://paperpile.com/b/QCn3le/q6Bqr) <http://dx.doi.org/10.1007/s00335-004-2380-6>

[3. Griffiths-Jones S, Grocock RJ, van Dongen S, Bateman A, Enright AJ. miRBase: microRNA sequences, targets and gene nomenclature. Nucleic Acids Res [Internet]. 2006;34:D140–4. Available from:](http://paperpile.com/b/QCn3le/FamDl) <http://dx.doi.org/10.1093/nar/gkj112>

[4. Dowell R, Odell A, Richmond P, Malmer D, Halper-Stromberg E, Bennett B, et al. Genome characterization of the selected long- and short-sleep mouse lines. Mamm Genome [Internet]. 2016;27:574–86. Available from:](http://paperpile.com/b/QCn3le/k8SED) <http://dx.doi.org/10.1007/s00335-016-9663-6>

[5. Yang H, Wang JR, Didion JP, Buus RJ, Bell TA, Welsh CE, et al. Subspecific origin and haplotype diversity in the laboratory mouse. Nat Genet [Internet]. 2011;43:648–55. Available from:](http://paperpile.com/b/QCn3le/CVsKu) <http://dx.doi.org/10.1038/ng.847>

[6. Cox A, Ackert-Bicknell CL, Dumont BL, Ding Y, Bell JT, Brockmann GA, et al. A new standard genetic map for the laboratory mouse. Genetics [Internet]. 2009;182:1335–44. Available from:](http://paperpile.com/b/QCn3le/pipQb) <http://dx.doi.org/10.1534/genetics.109.105486>

[7. McKenna A, Hanna M, Banks E, Sivachenko A, Cibulskis K, Kernytsky A, et al. The Genome Analysis Toolkit: a MapReduce framework for analyzing next-generation DNA sequencing data. Genome Res [Internet]. 2010;20:1297–303. Available from:](http://paperpile.com/b/QCn3le/H4aLy) <http://dx.doi.org/10.1101/gr.107524.110>

[8. Köster J, Rahmann S. Snakemake--a scalable bioinformatics workflow engine. Bioinformatics [Internet]. 2012;28:2520–2. Available from:](http://paperpile.com/b/QCn3le/6Mt43) <http://dx.doi.org/10.1093/bioinformatics/bts480>

[9. Baras AS, Mitchell CJ, Myers JR, Gupta S, Weng L-C, Ashton JM, et al. miRge - A Multiplexed Method of Processing Small RNA-Seq Data to Determine MicroRNA Entropy. PLoS One [Internet]. Public Library of Science; 2015 [cited 2017 Mar 30];10:e0143066. Available from:](http://paperpile.com/b/QCn3le/olQdN) <http://journals.plos.org/plosone/article/file?id=10.1371/journal.pone.0143066&type=printable>

[10. Quek C, Jung C-H, Bellingham SA, Lonie A, Hill AF. iSRAP - a one-touch research tool for rapid profiling of small RNA-seq data. J Extracell Vesicles [Internet]. 2015;4:29454. Available from:](http://paperpile.com/b/QCn3le/Vol15) <http://dx.doi.org/10.3402/jev.v4.29454>

[11. Friedländer MR, Mackowiak SD, Li N, Chen W, Rajewsky N. miRDeep2 accurately identifies known and hundreds of novel microRNA genes in seven animal clades. Nucleic Acids Res [Internet]. 2012;40:37–52. Available from:](http://paperpile.com/b/QCn3le/hFJOu) <http://dx.doi.org/10.1093/nar/gkr688>

[12. Sun Z, Evans J, Bhagwate A, Middha S, Bockol M, Yan H, et al. CAP-miRSeq: a comprehensive analysis pipeline for microRNA sequencing data. BMC Genomics [Internet]. 2014;15:423. Available from:](http://paperpile.com/b/QCn3le/Yaq0V) <http://dx.doi.org/10.1186/1471-2164-15-423>

[13. Langmead B, Trapnell C, Pop M, Salzberg SL. Ultrafast and memory-efficient alignment of short DNA sequences to the human genome. Genome Biol [Internet]. 2009;10:R25. Available from:](http://paperpile.com/b/QCn3le/42ycK) <http://dx.doi.org/10.1186/gb-2009-10-3-r25>

[14. Vitsios DM, Enright AJ. Chimira: analysis of small RNA sequencing data and microRNA modifications. Bioinformatics [Internet]. 2015;31:3365–7. Available from:](http://paperpile.com/b/QCn3le/4WYY4) <http://dx.doi.org/10.1093/bioinformatics/btv380>

[15. Johnson M, Zaretskaya I, Raytselis Y, Merezhuk Y, McGinnis S, Madden TL. NCBI BLAST: a better web interface. Nucleic Acids Res [Internet]. 2008;36:W5–9. Available from:](http://paperpile.com/b/QCn3le/nnDAx) <http://dx.doi.org/10.1093/nar/gkn201>

[16. Zhang Y, Xu B, Yang Y, Ban R, Zhang H, Jiang X, et al. CPSS: a computational platform for the analysis of small RNA deep sequencing data. Bioinformatics [Internet]. 2012;28:1925–7. Available from:](http://paperpile.com/b/QCn3le/hv2jj) <http://dx.doi.org/10.1093/bioinformatics/bts282>

[17. Li R, Yu C, Li Y, Lam T-W, Yiu S-M, Kristiansen K, et al. SOAP2: an improved ultrafast tool for short read alignment. Bioinformatics [Internet]. 2009;25:1966–7. Available from:](http://paperpile.com/b/QCn3le/Jepy3) <http://dx.doi.org/10.1093/bioinformatics/btp336>

[18. Langmead B, Salzberg SL. Fast gapped-read alignment with Bowtie 2. Nat Methods [Internet]. 2012;9:357–9. Available from:](http://paperpile.com/b/QCn3le/H4oKE) <http://dx.doi.org/10.1038/nmeth.1923>

[19. Quinlan AR, Hall IM. BEDTools: a flexible suite of utilities for comparing genomic features. Bioinformatics [Internet]. 2010;26:841–2. Available from:](http://paperpile.com/b/QCn3le/DVswa) <http://dx.doi.org/10.1093/bioinformatics/btq033>

[20. Hackenberg M, Rodríguez-Ezpeleta N, Aransay AM. miRanalyzer: an update on the detection and analysis of microRNAs in high-throughput sequencing experiments. Nucleic Acids Res [Internet]. 2011;39:W132–8. Available from:](http://paperpile.com/b/QCn3le/WMOek) <http://dx.doi.org/10.1093/nar/gkr247>

[21. Wang W-C, Lin F-M, Chang W-C, Lin K-Y, Huang H-D, Lin N-S. miRExpress: analyzing high-throughput sequencing data for profiling microRNA expression. BMC Bioinformatics [Internet]. 2009;10:328. Available from:](http://paperpile.com/b/QCn3le/bYS6J) <http://dx.doi.org/10.1186/1471-2105-10-328>

[22. Smith TF, Waterman MS. Identification of common molecular subsequences. J Mol Biol [Internet]. 1981;147:195–7. Available from:](http://paperpile.com/b/QCn3le/XlmSH) <https://www.ncbi.nlm.nih.gov/pubmed/7265238>

[23. Ronen R, Gan I, Modai S, Sukacheov A, Dror G, Halperin E, et al. miRNAkey: a software for microRNA deep sequencing analysis. Bioinformatics [Internet]. 2010;26:2615–6. Available from:](http://paperpile.com/b/QCn3le/hpHHD) <http://dx.doi.org/10.1093/bioinformatics/btq493>

[24. Li H, Durbin R. Fast and accurate short read alignment with Burrows-Wheeler transform. Bioinformatics [Internet]. 2009;25:1754–60. Available from:](http://paperpile.com/b/QCn3le/RTGrW) <http://dx.doi.org/10.1093/bioinformatics/btp324>

[25. Paşaniuc B, Zaitlen N, Halperin E. Accurate estimation of expression levels of homologous genes in RNA-seq experiments. J Comput Biol [Internet]. 2011;18:459–68. Available from:](http://paperpile.com/b/QCn3le/RsXAL) <http://dx.doi.org/10.1089/cmb.2010.0259>

[26. Wu J, Liu Q, Wang X, Zheng J, Wang T, You M, et al. mirTools 2.0 for non-coding RNA discovery, profiling, and functional annotation based on high-throughput sequencing. RNA Biol [Internet]. 2013;10:1087–92. Available from:](http://paperpile.com/b/QCn3le/LE4Pq) <http://dx.doi.org/10.4161/rna.25193>

[27. Capece V, Garcia Vizcaino JC, Vidal R, Rahman R-U, Pena Centeno T, Shomroni O, et al. Oasis: online analysis of small RNA deep sequencing data. Bioinformatics [Internet]. 2015;31:2205–7. Available from:](http://paperpile.com/b/QCn3le/Pl9uJ) <http://dx.doi.org/10.1093/bioinformatics/btv113>

[28. Dobin A, Davis CA, Schlesinger F, Drenkow J, Zaleski C, Jha S, et al. STAR: ultrafast universal RNA-seq aligner. Bioinformatics [Internet]. 2013;29:15–21. Available from:](http://paperpile.com/b/QCn3le/n3HeD) <http://dx.doi.org/10.1093/bioinformatics/bts635>

[29. Liao Y, Smyth GK, Shi W. featureCounts: an efficient general purpose program for assigning sequence reads to genomic features. Bioinformatics [Internet]. 2014;30:923–30. Available from:](http://paperpile.com/b/QCn3le/NfXZR) <http://dx.doi.org/10.1093/bioinformatics/btt656>

[30. Müller S, Rycak L, Winter P, Kahl G, Koch I, Rotter B. omiRas: a Web server for differential expression analysis of miRNAs derived from small RNA-Seq data. Bioinformatics [Internet]. 2013;29:2651–2. Available from:](http://paperpile.com/b/QCn3le/avbxG) <http://dx.doi.org/10.1093/bioinformatics/btt457>

[31. Gupta V, Markmann K, Pedersen CNS, Stougaard J, Andersen SU. shortran: a pipeline for small RNA-seq data analysis. Bioinformatics [Internet]. 2012;28:2698–700. Available from:](http://paperpile.com/b/QCn3le/uKsvM) <http://dx.doi.org/10.1093/bioinformatics/bts496>

[32. Guillermo Barturen Antonio Rueda Maarten Hamberg Angel Alganza Ricardo Lebron Michalis Kotsyfakis Bu-Jun Shi Danijela Koppers-Lalic Michael Hackenberg. sRNAbench: profiling of small RNAs and its sequence variants in single or multi-species high-throughput experiments. Methods in Next Generation Sequencing [Internet]. 2014;1. Available from:](http://paperpile.com/b/QCn3le/8pqex) <https://www.degruyter.com/view/j/mngs.2014.1.issue-1/mngs-2014-0001/mngs-2014-0001.xml>
